# Jitter Evaluation in Distant and Adjacent Muscles after Botulinum Neurotoxin Type A Injection in 78 Cases

**DOI:** 10.3390/toxins12090549

**Published:** 2020-08-27

**Authors:** Joao Aris Kouyoumdjian, Carla Renata Graça, Fabio Nazare Oliveira

**Affiliations:** 1 Laboratório Investigação Neuromuscular (LIN), Faculdade Estadual Medicina de São José do Rio Preto (FAMERP), São José do Rio Preto SP 15090-000, Brazil; carlarenata@famerp.br; 2 Departamento de Ciências Neurológicas, Fundação Faculdade Regional de Medicina São José do Rio Preto (FUNFARME), São José do Rio Preto SP 15090-000, Brazil; fabionazare@terra.com.br

**Keywords:** single-fiber electromyography, jitter, botulinum neurotoxin type A, movement disorders, myasthenia gravis, neuromuscular junction

## Abstract

To study the jitter parameters in the distant (DM) and the adjacent muscle (AM) after botulinum neurotoxin type A (BoNT/A) injection in 78 patients, jitter was measured by voluntary activation in DM (*n* = 43), and in AM (*n* = 35). Patients were receiving BoNT/A injections as a treatment for movement disorders. Mean age 65.1 years (DM) and 61.9 years (AM). The mean jitter was abnormal in 13.9% (maximum 41.4 µs) of DM, and 40% (maximum 43.7 µs) of AM. Impulse blocking was sparse. We found no correlation of the mean jitter to age, BoNT/A most recent injection (days/units), number of muscles injected, total BoNT/A units summated, number of total BoNT/A sessions, beta-blockers/calcium channel blockers use, and cases with local spread symptoms such as eyelid drop/difficulty swallowing. Maximum mean jitter (41.4/43.7 µs) for DM/AM occurred 61 and 131 days since the most recent BoNT/A, respectively. The far abnormal mean jitter (32.6/36.9 µs) occurred 229 and 313 days since the most recent BoNT/A. We suggested that jitter measurement can be done after BoNT/A in a given muscle other than the injected one, after 8 (DM) and 11 (AM) months, with reference >33 µs and >37 µs, respectively.

## 1. Introduction

In 1977, Scott et at. [1], seeking a pharmacological alternative for strabismus correction, found an extraordinary benefit from a botulinum neurotoxin type A (BoNT/A) injection into the rectus muscles in adult rhesus monkeys. Since then, symptoms from many movement disorders have been greatly minimized from the BoNT/A injections. Botulinum neurotoxin high efficacy occurs because it has high specificity for skeletal and autonomic cholinergic nerves. It does not spread in high amounts outside the injection site, and it is poorly immunogenic [2,3].

*Clostridium botulinum* produces eight immunologically distinct serotypes (type A–H) that differ from each other by the nontoxic accessory protein (NAP) composition. For the clinical purpose, the BoNT/A is the most used and presented as onabotulinumtoxinA (ONA), abobotulinum toxin A (ABO), and incobotulinum toxin A (INCO) [4]. For pharmacological action, all should be cleaved from the NAP, except INCO that contains just the active 150-kD neurotoxin [4].

Botulinum neurotoxins enter the nerve terminals, where they cleave specifically to vesicle-associated membrane protein or synaptobrevin (VAMP), the synaptosomal associate protein of 25 kDa (SNAP25) or syntaxin [5]. The proteins VAMP, SNAP25, and syntaxin in neuroexocytosis are collectively termed SNARE (SNAP REceptors) proteins because they were isolated as a receptor complex of the Soluble NSF Attachment Protein (SNAP) [5]. All BoNTs are expressed by two polypeptide chains (light 50 kDa, and heavy 100 kDa) linked by an interchain disulfide bond. If the light chain, which is the active part of the toxin, is present within a nerve terminal, the newly synthesized SNARE protein will continue to be cleaved [5]. The BoNT/B, BoNT/D, BoNT/F, and BoNT/G cleave VAMP. The BoNT/A and BoNT/E cleave SNAP25, and the BoNT/C cleaves both SNAP25 and syntaxin, resulting in the inhibition of acetylcholine release and paralysis [3].

Blocking neuromuscular transmission with BoNT/A, despite no injury to the motor axon, induces chemodenervation with a massive axon sprouting and neuromuscular junction remodeling [6,7]. Botulinum neurotoxin chemodenervation mimics axotomy, including the interruption of neurotransmission, induction of motor axon sprouting and upregulation of regeneration-associated proteins [8]. In rats, within a few days of the BoNT/A blocking, sprouts of the motor axons grow out over the surface of the muscle [7]. Around two weeks later, non-junctional or ectopic clusters of high acetylcholine receptor density begin to be settled. The effect of BoNT/A is long-lasting because the toxin irreversibly blocks the function of SNAP-25 molecules. However, a remarkable feature of the BoNT/A effect is its reversibility [5]. Recovery mechanisms include the formation of new proteins and axonal sprouting [9]. The neuromuscular block produced by the BoNT/A reaches its maximum in five days and, after that, remains stable for several months. After about ten days of BoNT, fibrillation potentials (FP) appeared and persisted throughout 60 days. The electromyographic findings in chronic botulinum poisoning are almost identical with those found following motor denervation, but in the last, the FP appears four to five days after the operation [10].

It is well known that the local effect of BoNT/A intramuscular injection either clinically (muscle paralysis) or through single-fiber electromyography (SFEMG) demonstrates very high jitter and impulse blocking [11,12,13,14]. The BoNT/A injection also may spread nearby, causing weakness, as seen in cervical dystonia (dysphagia) and in blepharospasm and hemifacial spasm (eyelid drop), that usually takes a few days to clear up [15]. Another defined point is the adjacent, and distant BoNT/A effect detected only through SFEMG with high jitter without or with sparse impulse blocking [11,16,17,18,19,20,21,22,23,24,25,26]. In a few cases, indisputable signs and symptoms of pseudo-botulism, such as weakness, blurred vision, diplopia, and difficulty swallowing, can occur after inaccurate dose injection. Finally, in very few cases, BoNT/A can precipitate or worsen symptoms from an unknown myasthenia gravis (MG) [27,28,29].

The reliability of the SFEMG jitter parameters in patients investigating neuromuscular junction disorders after receiving a BoNT/A injection is a challenge for the clinical neurophysiologists. From a practical perspective, there are no jitter reference values related to the time elapsed after the most recent BoNT/A injection obtained in a large patient cohort. The purpose here was to study the SFEMG jitter parameters in non-injected adjacent muscles and distant muscles from a large cohort of patients (78 cases) that have been in treatment with BoNT/A mostly for movement disorders. The objective is to find out the most reliable reference jitter parameters for those patients who had already been injected with BoNT/A and came to the SFEMG for a neuromuscular junction disorder suspicion.

## 2. Results

### 2.1. Patients

One patient with Alzheimer’s disease was excluded from the study due to difficulties in getting spikes, remaining 78 cases. No patient complained of systemic symptoms like fatigue, blurred vision, or weakness, and they were very satisfied with the BoNT/A clinical results. Spread symptoms due to BoNT/A was far more common in cervical dystonia patients, where 57.1% complained of dysphagia (“difficulty swallowing”) that lasted about 2 weeks; one case (7.1%) had associated dysphonia. In cases of hemifacial spasm, eyelid drop occurred in 28.9% extending for a few days, and dry eye occurred in 6.7% of patients. In patients with blepharospasm, just one case (7.7%) reported an eyelid drop. We found no relationship between cases with spread symptoms to the mean jitter values. Accordingly, the mean jitter was normal in the distant muscle (ED) in 5/7 (71.4%), and in the adjacent muscle (*Frontalis*) in 11/17 (64.7%) of those cases.

Most patients have comorbidities, and the mean daily pills used was 2.5, ranging from 0 to 11. Hypertension (35), depression (19), diabetes (10), hypothyroidism (9), and any arthritis (4) were the main associate disorders described by patients. History of atheromatous related-disorders, like chronic coronaropathy and mild stroke, were more difficult to obtain from patients, but some used antiplatelet medication. Any antihypertensive drug plus diuretics and beta-blockers represented the most used daily pills (*n* = 70), followed by antidepressant drugs (*n* = 19), statins (*n* = 13), insulin, and metformin (*n* = 10), levothyroxine (*n* = 9), anticonvulsants (*n* = 7), and calcium channel blockers (*n* = 5). We checked specifically the relationship between the beta-blockers/calcium channel blockers to the mean jitter. From 22 patients that were on beta-blockers, 17 had normal and 5 an abnormal mean jitter. Of 5 patients in the use of calcium channel blockers, 4 had normal, and 1 had an abnormal mean jitter. So, we could not find any association that could act as a potential confusion factor.

The first group, to measure jitter parameters in a distant muscle, comprised 43 patients, age 65.1 ± 10.5 years (35 to 83), 70% female. The patients were in the regular outpatient schedule for the BoNT/A injections due to hemifacial spasm (22), blepharospasm (10), cervical dystonia (6), aesthetics (2), bruxism (1), focal dystonia in the upper limb (1) and spasticity (1). BoNT/A was injected into one muscle (7 patients), two muscles (4 patients), three muscles (30 patients), and five muscles (2 patients). The most frequent injected muscles were *Orbicularis Oculi* (33), *Risorius* (21), *Zygomaticus Major* (21), *Corrugator Supercilii* (7), *Sternocleidomastoideus* (6), *Splenius Capitis* (6), *Levator Scapulae* (6), *Orbicularis Oris* (2), *Procerus* (2), *Flexor Carpi Radialis* (2), *Trapezius* (1), *Masseter* (1), *Frontalis* (1), *Levator Nasalis* (1), *Platysma* (1), *Pectoralis Major* (1), *Biceps Brachii* (1), *Tibialis Posterior* (1), and *Extensor Pollicis Longus* (1). In 2 patients, BoNT/A injections were made in the limb, but in these cases, the distant muscle tested was the contralateral one. In the remaining 41 patients, the muscles injected with BoNT/A were in the face or neck.

The second group, to measure jitter parameters in an adjacent muscle, comprised 35 patients, age 61.9 ± 12.1 years (30 to 79), 77% female. The patients were in the regular outpatient schedule for the BoNT/A injections due to hemifacial spasm (23), cervical dystonia (6), blepharospasm (3), segmental dystonia (1), generalized dystonia (1), and cervical dystonia plus blepharospasm (1). Botulinum neurotoxin type A was injected into two muscles (5 patients), three muscles (25 patients), four muscles (4 patients), and five muscles (1 patient). The most frequent injected muscles were *Orbicularis Oculi* (28), *Zygomaticus Major* (24), *Risorius* (23), *Sternocleidomastoideus* (8), *Splenius Capitis* (8), *Levator Scapulae* (6), *Corrugator Supercilii* (4), *Trapezius* (3), and *Masseter* (1). In all cases, the muscles injected with BoNT/A were in the neck or the face.

The variables referring to the age, mean jitter, percentage of abnormal individual jitter values (“outliers”), the time elapsed (days) for SFEMG test since the first and most recent BoNT/A injection, the number of BoNT/A injections since the first one, the BoNT/A units injected per session, the total BoNT/A units summated since the first one, and the number of muscles injected per session are shown in Table 1 (distant group) and Table 2 (adjacent group). The table also shows the normality test for each one for the appropriate statistical tests.

The comparison between distant versus adjacent groups revealed no significant difference between age, the number of injections, the number of days from the first and the most recent injection of BoNT/A, BoNT/A units per injection, total BoNT/A units summed, and the number of muscles injected with BoNT/A per session (Table 3).

### 2.2. Single-Fiber Electromyography

**Figure 1:**
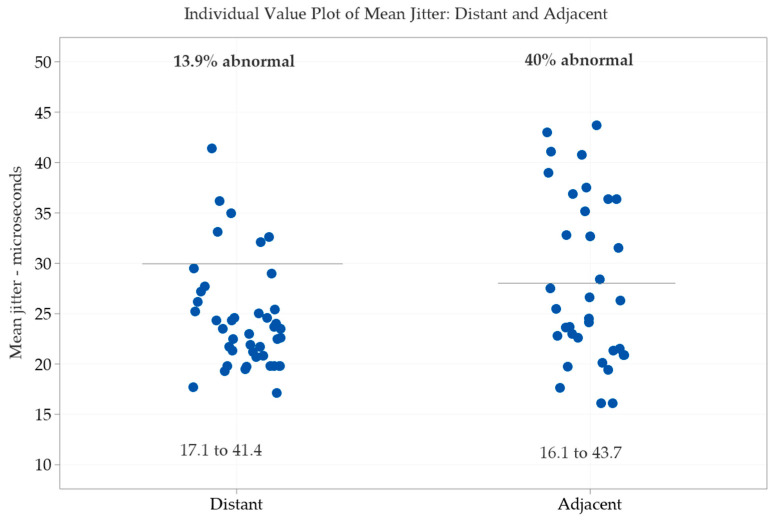
Individual value plot of the mean jitter in the distant (*n* = 43) compared to the adjacent group (*n* = 35). Observe the high proportion of increased jitter (40%) in the adjacent versus only 13.9% in the distant group. Distant muscle, mostly *Extensor Digitorum*; adjacent muscle, all *Frontalis*.

In the distant group (43 patients), the muscles studied were *Extensor Digitorum* (40), *Deltoideus* (2), and *Tibialis Anterior* (1). In the adjacent group (35 patients), the only muscle studied was the *Frontalis*. The comparison between distant versus adjacent groups revealed a significant difference between the percentage of abnormal individual jitter values (*p* = 0.0003), and the percentage of impulse blocking (*p* = 0.0383) (Table 3). The mean jitter in the distant group was abnormal in 13.9% (24.4 ± 5.28 µs), ranging from 17.1 to 41.1 µs, and impulse blocking was found in five cases (11.6%). The mean jitter in the adjacent group was abnormal in 40% (28 ± 8.2 µs), ranging from 16.1 to 43.7 µs, and impulse blocking was found 10 cases (28.6%) (Figure 1).

### 2.3. Correlation between Jitter and Variables

**Figure 2:**
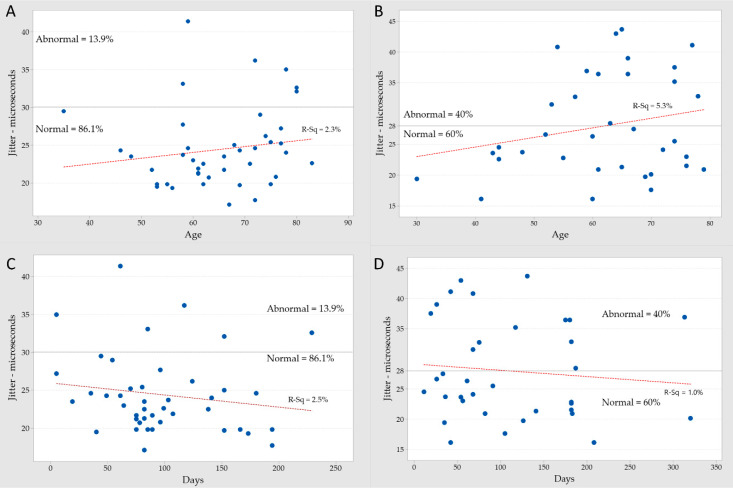
Correlation between the mean jitter and age in the most recent botulinum neurotoxin type A (BoNT/A) injection for the distant (**A**) and the adjacent muscle (**B**). Correlation between the mean jitter and days after the most recent BoNT/A injection in the distant (**C**) and the adjacent muscle (**D**).

The power of the correlation between the mean jitter values to some variables using a linear model and calculating the R-squared in percentiles were weak and ranged from 0% to 12.3% (Table 4). The correlation between age in the most recent BoNT/A injection versus mean jitter in the adjacent and distant muscle groups (A and B), and the number of days since the most recent BoNT/A injection versus mean jitter in the adjacent and distant muscle groups (C and D) are shown in Figure 2.

## 3. Discussion

Our results revealed that a relatively mild mean jitter increased in 13.9% of patients from the distant group, in which the BoNT/A was injected mostly in facial or neck muscle, and the jitter was measured in a limb muscle, mostly ED. The maximum mean jitter obtained was 41.4 µs (38% above the reference). For the patients in the adjacent group, in which the BoNT/A was injected mostly in facial or neck muscle and the jitter was measured in the *Frontalis* muscle, the mean jitter was increased by 40%. The maximum mean jitter obtained was 43.7 µs (56.1% above the reference). We found a statistically significant difference in impulse blocking between groups, with this being more frequent in the adjacent group. However, there was no correlation statistically significant for any variable-age, days from the most recent BoNT/A injection, BoNT/A units in the most recent injection, total number of injections, and total BoNT/A summated since the first injection to predict the mean jitter increase. We specify the extreme findings for the mean jitter (lowest/highest) in Table 5, and the longest time that we had done the SFEMG test after the last BoNT/A injection in Table 6.

Even though both groups were composed of relatively older patients (mean age 65.1 and 61.9 for the distant and adjacent group, respectively), with more comorbidities associated, and consequently more daily medicine use, we could not find any evidence of age bias for the jitter parameters. There was no difference in the mean jitter from those taking beta-blockers or calcium channel blockers. None of the patients presented a systemic effect that could be attributed to pseudo-botulism, nor any symptoms from BoNT/A intolerance. All patients were very satisfied with the clinical results. We could not find any association between the cases who presented symptoms from the local BoNT/A spread (mainly dysphagia for cervical dystonia and eyelid drop for hemifacial spasm and blepharospasm) with the mean jitter value in both groups.

We found fifteen papers related to the local or distant effect of BoNT/A in humans [11,12,13,14,15,16,17,18,19,20,21,23,24,26,30]. The comparison was not an easy task due to the number of patients or healthy subjects, muscle injected, BoNT/A units used, time to do the SFEMG test, selection of patients (asymptomatic, fatigue or pseudo-botulism), and some variability in the adjacent or distant muscles studied for jitter parameters. Jitter parameters were studied in the same muscle injected in healthy subjects in a total of 102 muscles (*Abductor Digiti Minimi*, *Extensor Digitorum Brevis*, *Extensor Digitorum*, and *Tibialis Anterior*) [13,14,21], and also in the same muscle injected from patients (*Orbicularis Oculi*) in a total of 18 muscles [11,12]. The electrophysiological findings were reduced CMAP’s amplitude (nadir at three weeks), no decrement at a slow frequency, no significant increment at 20 Hz (less than 10%), FP more frequently found within the first six weeks [31], high jitter increase from 2 weeks until 8 weeks (89 to more than 300 µs), frequent impulse blocking, and no significative increase in fiber density [32]. The findings mentioned above were typical for chemodenervation and sometimes took many months to restore to normal.

We had selected reports in which the jitter parameters were studied in the adjacent or distant muscles from injected muscles or regions in healthy subjects or patients, provided that they do not have symptoms (fatigue or pseudo-botulism). The BoNT/A was injected into *Orbicularis Oculi*, glabellar region (*Corrugator Supercillii* and *Procerus* muscles), neck region, intragastric, detrusor, and various muscles in the face. The adjacent or distant muscle jitter studies were done mostly in *Extensor Digitorum* (87) and *Orbicularis Oculi* (32) [11,15,16,17,18,19,20,24,26,30]. It is likely that the ED muscles studied in the cases from Ruet et al. (2015) were the same as those described by Schnitzler et al. [24] As a whole, the mean jitter values were increased in distant muscle from none to 80%. For the ED muscle, the mean jitter varied from 27 to 65 µs between 2 to 8 weeks since BoNT/A injection. For the *Orbicularis Oculi* muscle, 25.9 to 31.3 µs, between 2 to 8 weeks. For the *Biceps Brachii* muscle, 38 to 66 µs, between 4 to 6 weeks. No or rare impulse blocking was described. We consider the findings of Garner et al. [20] probably biased. They found remarkably increased jitter at week 4 (156 µs) with 100% impulse blocking in the *Extensor Digitorum Brevis* muscle, which frequently has neurogenic findings.

The significant decline of CMAP amplitude, of both the *Masseter* and the *Orbicularis Oris* muscles after BoNT/A injection in the *Orbicularis Oculi* muscle suggests local instead of spreading axonal diffusion [33]. It also has been demonstrated that BoNT/A diffused through the fascia to the adjacent muscles [34]. Alimohammad et al. [15] emphasizes the physical spread of BoNT/A through small intramuscular vessels from the glabellar injection sites to the *Orbicularis Oculi* muscle. However, even in this well-controlled study, not all subjects had jitter values increased after two weeks since the injection. Garner et al. [20] did not find a correlation between the units of BoNT/A and the SFEMG findings. In disagreement, Girlanda et al. [19] concluded a BoNT/A dose-dependent effect on neuromuscular transmission in distant muscles. Sanders et al. [11] were more cautious about answering this issue and concluded that the local and distant effects of BoNT/A are “probably” dose-related. Our study did not find any correlation between the jitter parameters and the BoNT/A units (dose) used. We did not find a relationship with larger BoNT/A being expected to add distant jitter, as pointed out by Sanders et al. [11] The distant increased jitter is usually attributed to a systemic blood BoNT/A spread. In the present study, we could speculate an individual factor for BoNT/A reaching the muscle target on a distant limb since we find just 13.9% of patients with increased jitter. For the adjacent group, the “spread factor” is probably the operator’s expertise to inject BoNT/A to the correct muscle motor point. As already mentioned, we had not found any isolated variable that could predict any direction to this question.

For the practical electrodiagnostic perspective for carrying out the SFEMG test for a MG suspicion, in a patient already injected with BoNT/A, we suggest reviewing the following rules. The major (and rare) systemic motor-autonomic symptoms related to the BoNT/A injection is the pseudo-botulism presented with diplopia, dysphagia, and dysarthria followed by a descending pattern of weakness affecting the upper limbs, then lower limbs, and in severe cases, respiratory muscle weakness, associated with blurred vision, constipation, dry mouth, postural hypotension, urinary retention, and pupillary abnormalities. The significant motor-autonomic symptoms related to the BoNT/A adjacent spread are dysphagia (it can extend for many weeks after neck muscles BoNT/A injection for cervical dystonia), eyelid drop (can take just a few days after *Orbicularis Oculi* BoNT/A injection), and dry eye. The unspecific fatigue symptom after BoNT/A should be seen with caution and clearly distinguished from the fluctuating weakness. If a given patient had a mean jitter greater than 37 µs for the *Frontalis* muscle (voluntary activation), and more than 11 months after the most recent BoNT/A injection, the increased jitter would probably be related to the MG. If a given patient had the mean jitter greater than 33 µs for the *Extensor Digitorum* muscle (voluntary activation), more than eight months after the most recent BoNT/A injection, the increased jitter would probably be related to the MG. If a given patient had a mean jitter higher than 43 µs (ED) and 45 µs (*Frontalis* muscle) at any time, it will probably not be related to BoNT/A. The SFEMG test should never be done in the injected muscle, at any time.

There may be some possible minor limitations in this study. We did not measure the jitter parameters of the same patient at regular intervals after the BoNT/A injection due to the difficulty of having multiple SFEMG tests. However, this limitation was compensated by a large number of patients. Another minor limitation was the absence of reported CNE jitter reference for some muscles barely studied for the neuromuscular transmission suspicion disorders, as *Deltoideus* and *Tibialis Anterior* muscles. We consider it not a significant problem since the parameter variation for the reference jitter values obtained through CNE is much less than for SFE, and was used in 3 out of 43 muscles.

## 4. Conclusions

In conclusion, we found no cases with pseudo-botulism or fatigue symptoms. The percentage of cases with spread-related symptoms (dysphagia and eyelid drop) is the same as already reported. We found a jitter increase in a distant muscle from BoNT/A injection in 13.9% of 43 patients, the highest value being 41.4 µs (limit 30 µs). For the adjacent muscle from BoNT/A injection, we found a jitter increase in 40% of 35 patients, the highest value being 43.7 µs (limit 28 µs). There was sparse impulse blocking. There was no correlation between the mean jitter and several variables-days/units after the most recent BoNT/A injection, the number of muscles injected per session, the total number of BoNT/A sessions, total units summated since the first one, MUAP’s amplitude, daily use of beta-blockers/calcium channel blockers, cases with symptoms from the local spread, and age. We presume that personal factors could be involved in the distant group and operator injection precision for the adjacent group. For electrodiagnostic practice, we suggested that jitter measurement can be analyzed after BoNT/A in a given muscle other than the injected, after eight (distant) and eleven (adjacent) months, with reference limit >33 µs for and >37 µs, respectively.

## 5. Materials and Methods

### 5.1. Patients

Between March 2018 to October 2019, 79 patients aged 30–83 years old, of both sexes, who routinely were injected with BoNT/A for movement disorders at the specific outpatient clinic of the Lucy Montoro Rehabilitation Center, Sao Jose do Rio Preto State Medical School (FAMERP), were recruited and invited to participate in the study. All cases were under the supervision of one of the authors (FO), the board-neurologist in charge of the diagnosis, and intramuscular injections of BoNT/A. Demographic data (sex and age), clinical data (diagnosis of movement disorder), comorbidities known to patients or described in the electronic medical records, drugs in continuous use, BoNT/A data (type, brand, the amount injected, periodicity, injected muscles, systemic adverse reactions, local adverse reaction, and result), time in days from the most recent BoNT/A injection to the SFEMG test, time in days from the first BoNT/A injection until the SFEMG test, and total dose (summated units) of BoNT/A until the SFEMG tests were done. The BoNT/A dose (units) was related to the muscle size. Abobotulinum toxin A (ABO; Dysport^®^/Ipsen Limited, Slough Berkshire, UK) was used in 77 patients. Only one patient has used three different BoNT/A injections over the years: Abobotulinum toxin A (ABO; Dysport^®^/Ipsen Limited, Slough Berkshire, UK), Incobotulinum toxin A (INCO; Xeomin^®^/Bocouture, Merz Pharmaceuticals GmbH, Frankfurt, Germany), and OnabotulinumtoxinA (ONA; Botox^®^/Vistabel, Allergan Inc., Irvine, CA, USA). The dilution was the same for all, 20U for every 0.1 mL.

### 5.2. Inclusion Criteria

Patients with regular BoNT/A injections in the neck or facial muscles to measure the jitter in distant or adjacent muscle. Patients with regular BoNT/A injections in limb muscles to measure jitter in distant and contralateral muscle. Subjects with regular BoNT/A injections for aesthetic purposes in facial muscles provided that the jitter’s measurement was done on a distant limb muscle.

### 5.3. Exclusion Criteria

Myasthenia gravis patients. Patients with previously confirmed neuromuscular diseases. Any weakness from known or unknown causes. Electromyographic evidence of active denervation or chronic reinnervation in the SFEMG tested muscle. Patients under 18 years of age. Patients without conditions to sustain minimal voluntary activation in the muscle studied. Patients in the use of anticoagulants.

### 5.4. Single-Fiber Electromyography

The SFEMG test to measure jitter parameters was performed at the Neuromuscular Investigation Laboratory at the FAMERP by just one of the authors (JK), a neurologist, and a clinical neurophysiologist who was board-certified. All patients were regularly scheduled for BoNT/A injections for movement disorders, mostly for hemifacial spasm, blepharospasm, and cervical dystonia (together, 91%); other disorders included bruxism, focal dystonia, generalized dystonia, spasticity, and aesthetics (together, 9%). The *Orbicularis Oculi*, *Zygomaticus Major*, *Risorius, Splenius Capitis*, *Sternocleidomastoideus*, *Levator Scapulae*, and *Corrugator Supercilii* muscles represented 91%. The *Trapezius*, *Orbicularis Oris*, *Masseter*, *Procerus*, *Flexor Carpi Radialis*, *Platysma*, *Frontalis, Levator Nasalis*, *Pectoralis Major*, *Biceps Brachii*, *Tibialis Posterior*, and *Extensor Pollicis Longus* muscles represented the remaining. Most of the patients had multiple muscles injected.

For the SFEMG test, patients were divided into two groups. In the first group (distant, 43 patients), the analysis was performed in a limb muscle, as follows, *Extensor Digitorum* (*n* = 40), *Deltoideus* (*n* = 2), and *Tibialis Anterior* (*n* = 1). In this group, the BoNT/A was injected in the neck or facial muscles (41 cases), in the forearm muscle (1 case being the SFEMG test performed in the opposite ED muscle) and in multiple muscles in one side of the body (1 case, being the SFEMG test performed in the opposing ED muscle). The choice of two other muscles instead of the ED was made when FPs were found, or the motor unit action potentials (MUAPs) revealed increased amplitude/duration (active denervation and chronic reinnervation, respectively) in the electromyography (EMG). In the second group (adjacent, 35 patients), the SFEMG test was performed in the *Frontalis* muscle in all cases. In these patients, the BoNT/A injection was performed in the neck or facial muscles, and not in the *Frontalis* muscle directly. The measurement of jitter parameters was done using the Natus™ Keypoint-Net (Middleton, WI, USA) or Natus™ UltraPro S100 (Middleton, WI, USA) machines, and an in-built software specifically developed for the SFEMG test. Before the SFEMG test, conventional non-quantitative EMG was performed in the chosen muscle to exclude neurogenic abnormalities.

The measurement of the jitter parameters by voluntary activation was performed using disposable concentric needle electrodes (CNE) of two brands: Ambu^®^ Neuroline Concentric, 25 mm × 0.30 mm (30 G), recording area 0.02 mm^2^ and, Natus™ Dantec^®^ DCN Disposable Concentric Needle Electrode, 25 mm × 0.30 mm (30 G), recording area 0.02 mm^2^. Both electrodes are routinely used and approved by the Brazilian Agency for Health Surveillance (ANVISA). The CNE was introduced into the chosen muscle. After minimal maintained voluntary contraction, the single fiber action potentials (SFAPs), or more appropriately, the “apparent single fiber action potentials” (ASFAPs) were recorded. Due to the use of disposable CNE, with a much larger recording area than the single fiber electrode, the probability of recording spikes from two or more muscle fibers increased; thus, the term ASFAP is better for defining these potentials. The low-frequency filter was set at 1 kHz to suppress the distant action potentials (APs) from the CNE recording area, passing only the APs with rapid rise time. Because the low-frequency filter reduces the amplitude of APs, we accepted only the ones with a minimum amplitude limit of 100 μV. Records were made after a minimum of three CNE insertions with radial advancement into the muscle. After slight movements of the electrode, a pair of ASFAPs was recorded, with both potentials belonging to the same motor unit; the trigger was put to the higher amplitude spike to get the temporal variation on the second smaller spike.

The jitter value was calculated as the mean consecutive differences (MCD) for each pair, ideally for 100 pairs; in some circumstances, we accepted a minimum of 30 pairs. The MCD variation was calculated by the “amplitude level”, i.e., by the temporal variation of the ascending lines of depolarization of the ASFAPs. Acceptable potentials were based on the criteria established in the literature [11], as follows: parallel depolarization ascending lines; similar shape in the superimposition of potentials; absence of summation characterized by notches and shoulders in the APs depolarization lines; a regular peak of the APs. Small amplitude variations were tolerated. We excluded ASFAP pairs without clear separation between them, records with less than 30 ASFAP pairs, ASFAP pairs with interpotential intervals (IPI) greater than 4 ms to avoid the effect of velocity recovery function (VRF), or when the shape of potentials did not remain constant in consecutive discharges. The classic SFAPs presents rise-time < 300 μs and the same shape/amplitude in consecutive discharges, quickly confirmed in the superimposition of at least ten potentials and, APs pairs must have a clear baseline separation.

The test conclusion was defined by the mean of 20 different MCDs for the reference value of 30 μs for the ED, *Deltoideus* and *Tibialis Anterior* muscles, and 28 µs for the *Frontalis* muscle [35]. Although the multicenter study for the new reference values for CNE jitter parameters did not contemplate large muscles other than the *Extensor Digitorum*, we use its reference for the *Deltoideus* muscle (use here in 2 cases) and the *Tibialis Anterior* muscle (used here in 1 case). The mean value of the sorted differences (MSD) was also obtained. MSD calculates the consecutive differences according to the frequency of discharge of the potential pairs. When the inter-discharge interval (IDI) variation is larger than 4 ms and not constant, there may be interference from the VRF effect. The mean jitter was calculated by the mean MCD or MSD, which had the lowest value for each pair. Mean jitter values higher than 150 μs were fixed at this value, to minimize the bias due to the VRF effect; the higher the jitter, the greater the probability of impulse blocking, consequently further artificially adding jitter. The other way for the test conclusion was calculated as the percentage of the individual MCDs above the reference limit (43 μs); when more than 10% of the ASFAP pairs, i.e., at least 3 out of 20, have values higher than 43 μs for the ED, *Deltoideus* and *Tibialis Anterior* muscles and, 38 µs for the *Frontalis* muscle, the test was abnormal. Impulse blocking was considered present or absent, regardless of the percentage in each pair. The skin temperature was maintained at more than 30 °C.

### 5.5. Statistics

Descriptive statistics calculated the mean jitter (MCD) values of the 78 cases and all other parameters: mean and standard deviation for normal or Gaussian distribution or median/percentage for non-normal distribution. An Anderson–Darling normality test was used. The Student’s *t*-test made the comparison of parametric variables with a significance of 0.05. The comparison of nonparametric variables was made by the Mann–Whitney nonparametric test (*U*-test) to verify the medians’ equality. Correlation of the mean jitter values (MCD) with variables was made with a linear model, calculating the R-squared in percentiles ranging from 0 to 100% to show the correlation power of jitter in the regression line.

### 5.6. Ethics

The study was in accord with the Helsinki Declaration of 1975 and approved by the ethics committee of the Faculdade de Medicina de São José do Rio Preto, São Paulo, Brazil, where the SFEMG tests were performed. Certificate of Presentation for Ethical Consideration (CAAE) approved number 83336818.0.0000.5415 on 22 March 2018. All patients signed informed consent.

## Figures and Tables

**Table 1:** Age, some jitter parameters, motor unit action potentials (MUAP) amplitude from the tested muscle, and some botulinum neurotoxin type A (BoNT/A) variables in the distant muscle group (43 patients).

Variables	Normality-Test	Mean	SD	Min	Q1	Median	Q3	Max
Age	Yes	65.12	10.47	35				83
Mean jitter (µs)	No			17.1	20.7	23.5	26.2	41.4
Abnormal individual jitter values (%)	No			0	0	5	5	30
Blocking (%)	No			0	0	0	0	5
BoNT/A first (days)	No			0	1099	1743	2331	6575
BoNT/A last (days)	No			5	64	85	138	229
Number of injections	No			1	9	13	18	30
Units per injection	No			30	60	60	80	600
Units total since first	No			60	660	880	1440	9600
Number muscles injected	No			1	2	3	3	5

MIPI, mean inter-potential interval; SD, standard-deviation; Min, Minimum; Max, Maximum; Q1, Interquartile Range 1; Q3, Interquartile Range 3.

**Table 2:** Age, some jitter parameters, motor unit action potentials (MUAP) amplitude from the muscle, and some botulinum neurotoxin type A (BoNT/A) variables in the adjacent muscle group (35 patients).

Variables	Normality-Test	Mean	SD	Min	Q1	Median	Q3	Max
Age	Yes	61.91	12.08	30				79
Jitter (µs)	No			16.1	21.3	25.5	36.4	43.7
Abnormal individual jitter values (%)	No			0	5	10	25	70
Blocking (%)	No			0	0	0	5	15
BoNT/A first (days)	No			238	1190	2050	3003	4365
BoNT/A last (days)	No			11	42	82	182	320
Number of injections	Yes	15.7	8.02	3		9		28
Units per injection	No			45	60	60	200	550
Units total since first	No			180	765	1200	1620	
Number muscles injected	No			2	3	3	3	5

MIPI, mean inter-potential interval; SD, standard-deviation; Min, Minimum; Max, Maximum; Q1, Interquartile Range 1; Q3, Interquartile Range 3.

**Table 3:** Comparison between age, some jitter parameters, and some botulinum neurotoxin type A (BoNT/A) variables, from the distant and adjacent group.

Variables	Distant vs. Adjacent	*t*-Test	*U*-Test	Significance
Age	*p* = 0.2138	Yes		No
BoNT/A first (days)	*p* = 0.1689		Yes	No
BoNT/A last (days)	*p* = 0.9661		Yes	No
BoNT/A number of injections	*p* = 0.2785	Yes		No
BoNT/A Units per injection	*p* = 0.9633		Yes	No
BoNT/A Units total	*p* = 0.1153		Yes	No
Number of muscles injected	*p* = 0.0582		Yes	No
Mean jitter	*p* = 0.0723		Yes	No
Abnormal individual jitter values (%)	*p* = 0.0003		Yes	Yes
Impulse blocking (%)	*p* = 0.0383		Yes	Yes

MIPI, mean inter-potential interval; *t*-test, Student’s *t*-test; *U*-test, Mann–Whitney test.

**Table 4:** Correlation between the mean jitter values with some variables with a linear model (R-squared in percentiles) to show the correlation power in the regression line (0% no correlation; 100% strongest correlation).

Variables	Distant Muscle	Adjacent Muscle	Power
Mean jitter vs. BoNT/A units per injection	12.8%	5.9%	weak
Mean jitter vs. total BoNT/A units summated	11.4%	3.3%	weak
Mean jitter vs. days after last BoNT/A injection	2.5%	1.0%	weak
Mean jitter vs. age in the last BoNT/A injection	2.3%	5.3%	weak
Mean jitter vs. MUAP amplitude	1.9%	0.0%	weak
Mean jitter vs. number of injected muscles	1.4%	4.6%	weak
Mean jitter vs. days after first BoNT/A injection	0.8%	0.0%	very weak

BoNT/A, botulinum neurotoxin type A; MUAP, motor unit action potentials.

**Table 5:** The highest and lowest mean jitter related to age, sex, and the most recent botulinum neurotoxin type A (BoNT/A) injection (days and units), in the distant muscle (*Extensor Digitorum*), and in the adjacent muscle (*Frontalis*). M, male; F, female.

Variables	Distant	Distant	Adjacent	Adjacent
Lowest mean jittert		17.1 µs		16.1 µs
Highest mean jitter	41.4 µs		43.7 µs	
BoNT/A last injection	61 days	82 days	131 days	42 days
Units	600 U	30 U	90 U	550 U
Age/Sex	51 M	67 F	65 M	60 F

**Table 6:** The longest time for jitter measurements after botulinum neurotoxin type A (BoNT/A) injection related to age, sex, and days/units since the most recent BoNT/A, in the distant muscle (*Extensor Digitorum*), and in the adjacent muscle (*Frontalis*).

Variables	Distant	Distant	Adjacent	Adjacent
Lowest mean jitter		17.7 µs		20.1 µs
Highest mean jitter	32.6 µs		36.9 µs	
BoNT/A last injection	229 days	194 days	313 days	320 days
Units	60 U	60 U	400 U	45 U
Age/Sex	80 F	72 M	59 M	70 M
